# A study protocol for integrating outpatient services at the primary health care level as part of the universal health coverage benefit package within the national health insurance program of Pakistan through private health facilities

**DOI:** 10.3389/fpubh.2024.1293278

**Published:** 2024-03-12

**Authors:** Syed Khurram Azmat, Ellen Mpangananji Thom, Muhammad Arshad, Hasan Bin Hamza, Atiya Aabroo, Asma Balal, Muhammad Ali Awan, Faisal Rifaq, Nilmini Hemachandra, Uzma Qudsia

**Affiliations:** ^1^Marie Stopes Society, Karachi, Pakistan; ^2^AIPH - AAPNA Institute of Public Health, Jinnah Sindh Medical University, Karachi, Pakistan; ^3^World Health Organization, Islamabad, Pakistan; ^4^Federal Sehat Sahulat Program, Islamabad, Pakistan; ^5^Ministry of National Health Services, Regulations, and Coordination, Islamabad, Pakistan; ^6^World Health Organization, Yangon, Myanmar

**Keywords:** universal health coverage, health insurance, primary health care, outpatient services, private sector, general practitioners, Pakistan

## Abstract

**Introduction and aim:**

Pakistan has a mixed-health system where up to 60% of health expenditures are out of pocket. Almost 80% of primary healthcare (PHC) facilities are in the private sector, which is deeply embedded within the country’s health system and may account for the unaffordability of healthcare. Since 2016, the existing national health insurance program or Sehat Sahulat Program (SSP), has provided invaluable coverage and financial protection to the millions of low-income families living in Pakistan by providing inpatient services at secondary and tertiary levels. However, a key gap is the non-inclusion of outpatient services at the PHC in the insurance scheme. This study aims to engage a private provider network of general practitioners in select union councils of Islamabad Capital Authority (ICT) of Pakistan to improve access, uptake, and satisfaction and reduce out-of-pocket expenditure on quality outpatient services at the PHC level, including family planning and reproductive health services.

**Methods and analysis:**

A 24-month research study is proposed with a 12-month intervention period using a mixed method, two-arm, prospective, quasi-experimental controlled before and after design with a sample of 863 beneficiary families from each study arm, i.e., intervention and control groups (*N* = 1726) will be selected through randomization at the selected beneficiary family/household level from four peri-urban Union Councils of ICT where no public sector PHC-level facility exists. All ethical considerations will be assured, along with quality assurance strategies. Quantitative pre/post surveys and third-party monitoring are proposed to measure the intervention outcomes. Qualitative inquiry with beneficiaries, general practitioners and policymakers will assess their knowledge and practices.

**Conclusion and knowledge contribution:**

PHC should be the first point of contact for accessing health services and appears to serve as a programmatic engine for universal health coverage (UHC). The research aims to study a service delivery model which harnesses the private sector to deliver an essential package of health services as outpatient services under SSP, ultimately facilitating UHC. Findings will provide a blueprint referral system to reduce unnecessary hospital admissions and improve timely access to healthcare. A robust PHC system can improve population health, lower healthcare expenditure, strengthen the healthcare system, and ultimately make UHC a reality.

## Introduction

### Problem statement

Achieving Universal health coverage (UHC) is one of the overarching targets of the 2030 agenda for Sustainable Development Goals (SDGs). Target 3.8 of Goal 3 aims to “achieve universal health coverage, including financial risk protection, access to quality essential health care services, and access to safe, effective, quality, and affordable essential medicines and vaccines for all” (1, 2). UHC is about ensuring that people have access to the health care they need, when and where they need them without suffering financial hardship and includes the full range of essential health services, from health promotion to prevention, treatment, rehabilitation, and palliative care (3).

A Lancet study of 153 countries reports that universal health coverage improves access to essential care and advances the population’s health, significantly benefiting the poorest segment (4). Global commitment to UHC is reflected in the first-ever UN high-level meeting on UHC convened in September 2019 that reaffirmed the political commitment to UHC under point 10 of the declaration (5). Access to affordable, quality primary healthcare (PHC) is the cornerstone of UHC. A strong primary health care system is easy and convenient access to a trusted service provider or a team of providers that operates in the local community and seeks to address all health problems of the entire population (6). Thus, population health will improve by integrating primary care services with public health, lowering overall healthcare expenditure over time, improving the healthcare system’s performance, and ensuring improved equity provision and access for everyone (7). Available global evidence points to the success of social protection models and health financing tools such as insurance, conditional/unconditional cash transfers, vouchers, etc., in addressing inequities in access to primary, secondary, and tertiary care services to the underserved. These are reported to be an emerging high-impact best practice in maternal, neonatal and child health (MNCH) and family planning (FP) service delivery (8–14). However, there are disparities in access to and affordability of healthcare services, especially PHC services. At least half of the people globally do not receive these services, and high **out-of-pocket (OOP) expenses** push about 100 million people annually into extreme poverty (2, 15). Providing access to PHC services to lower socio-economic groups will increase the use of healthcare services (16, 17).

### Country context and rationale

With an estimated population of over 216 million, Pakistan is the fifth most populous country in the world, where 35% of the total population still live under the US$3.20 per day poverty threshold (18). Access to health care services is even more challenging for those living below the poverty line and residing in rural, hard-to-reach geographic areas. More than 80% of primary health care is in the private sector, deeply embedded in the country’s healthcare system, resulting in high OOP expenditure by service seekers (19). Most private health expenditures are out-of-pocket payments, as the percentage of current health expenditures stands under 60% (20). In some areas, most people bear these expenditures by selling their assets, borrowing from others, ignoring the problem, and remaining untreated (21). While health is delivered through public and private providers, the share of the private sector is significantly larger than the public sector. In Pakistan, the equitable UHC for essential health services, especially for maternal, newborn and child health services related to promotion, prevention, and treatment coverage among the general and the most disadvantaged population, stands at the lowest in the South Asian region (22). While the public sector funds over 30% of health expenditure in Pakistan, the private sector accounts for over 70%, amounting to more than 470 billion PKR (USD 2.1 billion) annually (19), with the majority, as described earlier, being OOP expenditures - 47% of the total expenditure is allocated to medications, while 29% is designated for outpatient care. In a given year, 7.65% of households experience catastrophic health expenses exceeding 10% of their total household spending (23). Therefore, at the individual level, this can be translated into every person in Pakistan paying on average $89 annually for seeking essential health care at the point of use, and this depriving household spending annually pushes 1% of the households in the country below the $1.90 a day poverty line (24).

A key objective of the UHC Partnership (UHC-P) is to develop a comprehensive set of essential healthcare services (25). Pakistan became part of the “Phase 3–2016-2018” UHC Partnership (UHC-P) in 2018. The activities conducted under this partnership include governance, financing, service delivery, health workforce development, and health information system enhancement. However, certain areas such as medicines, non-communicable diseases (NCDs), communicable diseases, maternal and child health, antimicrobial resistance, and health security are not covered. Despite UHC-P not primarily focused on primary healthcare (PHC), the 2018 country reports indicated that numerous activities supported by UHC-P aligned with the modernized PHC concept. For example, Pakistan expanded its benefits package to include maternal and child health, certain communicable and non-communicable diseases and multi-sectoral health interventions for PHC and higher-level facilities (25, 26). Universal Health Coverage (UHC) stands as a global priority. It constitutes target 3.8 of the Sustainable Development Goals (SDG), which aims to achieve “financial risk protection, access to quality essential health-care services, and access to safe, effective, quality, and affordable essential medicines and vaccines for all,” attaining this objective necessitates enhancements in both population-level interventions and individual health services to foster health promotion and deliver preventive and therapeutic care (27). Monitoring progress toward this target will be facilitated by indicator 3.8.1, which focuses on the coverage of essential health services, and indicator 3.8.2, which centres on financial risk protection (28). To enhance the effectiveness of UHC, researchers have proposed indices measuring the coverage of essential health services, aiming to optimize the outcomes of UHC initiatives (29, 30).

Researchers have made progress toward measuring universal health coverage (UHC), but far less is known about the utilization and unit cost of services of health systems that will expand coverage (31). Some studies provided evidence that even in settings where progress has been made on UHC, deaths due to poor quality services represent a substantial challenge (32). From a financing standpoint, underperforming health systems reduce the returns on UHC investments. However, when coupled with investments in health system quality, expanding insurance can result in major health gains, as shown by Thailand, Rwanda, and Costa Rica, which have pursued this dual strategy and achieved substantial improvements in survival in child and maternal health 28-30 (33–35). Similar improvements in reducing catastrophic health expenditure and another impact on the progress of UHC were seen in studies from Russia, Mexico and Myanmar (36–38).

The Government of Pakistan launched one of the largest social protection initiatives as early as 2008, entitled Benazir Income Support Program (BISP), which provided unconditional cash transfers every quarter to the poorest using a BISP poverty scorecard (39). In 2015–2016, the Ministry of National Health Services, Regulations and Coordination (MoNHSRC), Government of Pakistan, in collaboration with Provincial Governments, launched the Prime Minister’s National Health Insurance Program (later renamed as Sehat Sahulat Programme - SSP), representing a national effort to achieve UHC by covering the cost of treatment for insured families to access services for a range of preventive, curable and promotive healthcare services (40). SSP provides financial protection to millions of insured poor households by providing them with in-patient services at the secondary and tertiary levels (41, 42). The scheme currently covers more than 37 million families, and their beneficiaries get to avail these services at the approved empanelled facilities living in Islamabad, Punjab and Khyber Pakhtunkhwa (KP) provinces, Azad Jammu Kashmir (AJK) and Gilgit Baltistan (GB).

Globally, outpatient visits have increased by more than half, and inpatient admissions have increased by more than two-thirds since 1990. Still, from the effectiveness and efficiency viewpoint, more research is needed, especially in the local context, on the utilization and unit costs of primary health services in the health systems that will expand coverage over time (31). A key gap in the current Sehat Sahulat Program is the absence of coverage for Outpatient (or OPD) services at the PHC level - which includes services related to sexual and reproductive health, maternal and child health, family planning, neonatal care, nutrition, adolescent health, non-communicable diseases, and infectious diseases (43). To reduce OOP expenses for accessing PHC, a financial benefits package for outpatient services must be implemented within the Pakistani government’s SSP program.

### Aim and objectives

This 24-month research study initiative aims to engage a private provider network in select union councils of Islamabad Capital Authority (ICT) of Pakistan to improve access, uptake, and satisfaction and reduce OOP expenditure on quality OPD services at the primary health care level, including family planning and sexual and reproductive health services.

The specific objectives of this intervention are:

Examine and document the determinants of outpatient services access and uptake.

Measure the changes in OOP expenditures, treatment outcomes, user satisfaction and quality of care.

Study and document health insurance outcomes using the capitation model intervention for modified outpatient EPHS services.

Generate evidence for the scale-up and delivery of integrated services as OPD services through SSP.

*Users, providers, and policymakers’ perspectives will be examined and documented.

## Materials and methods

### Conceptual model

**Figure 1:**
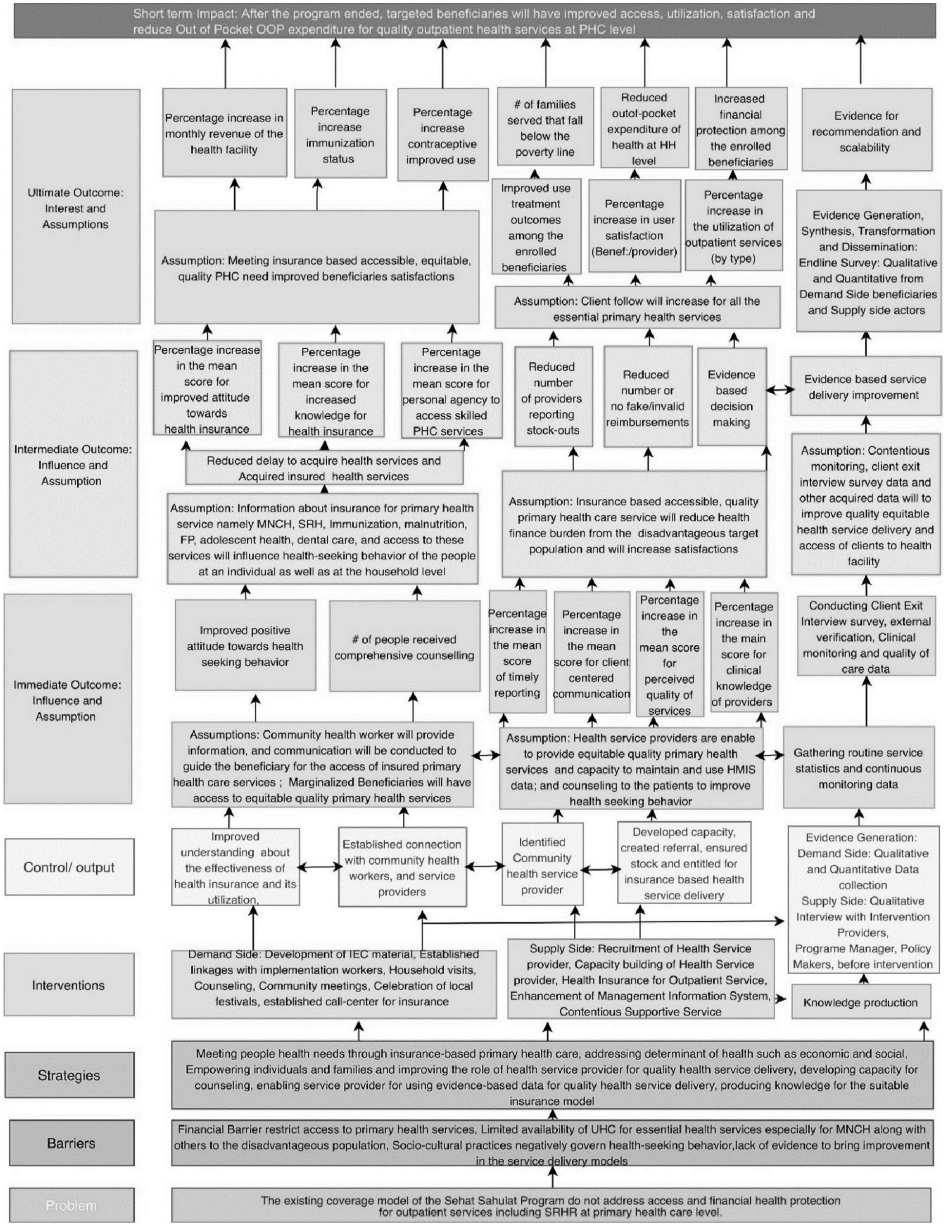
The theory of change.

**Figure 2:**
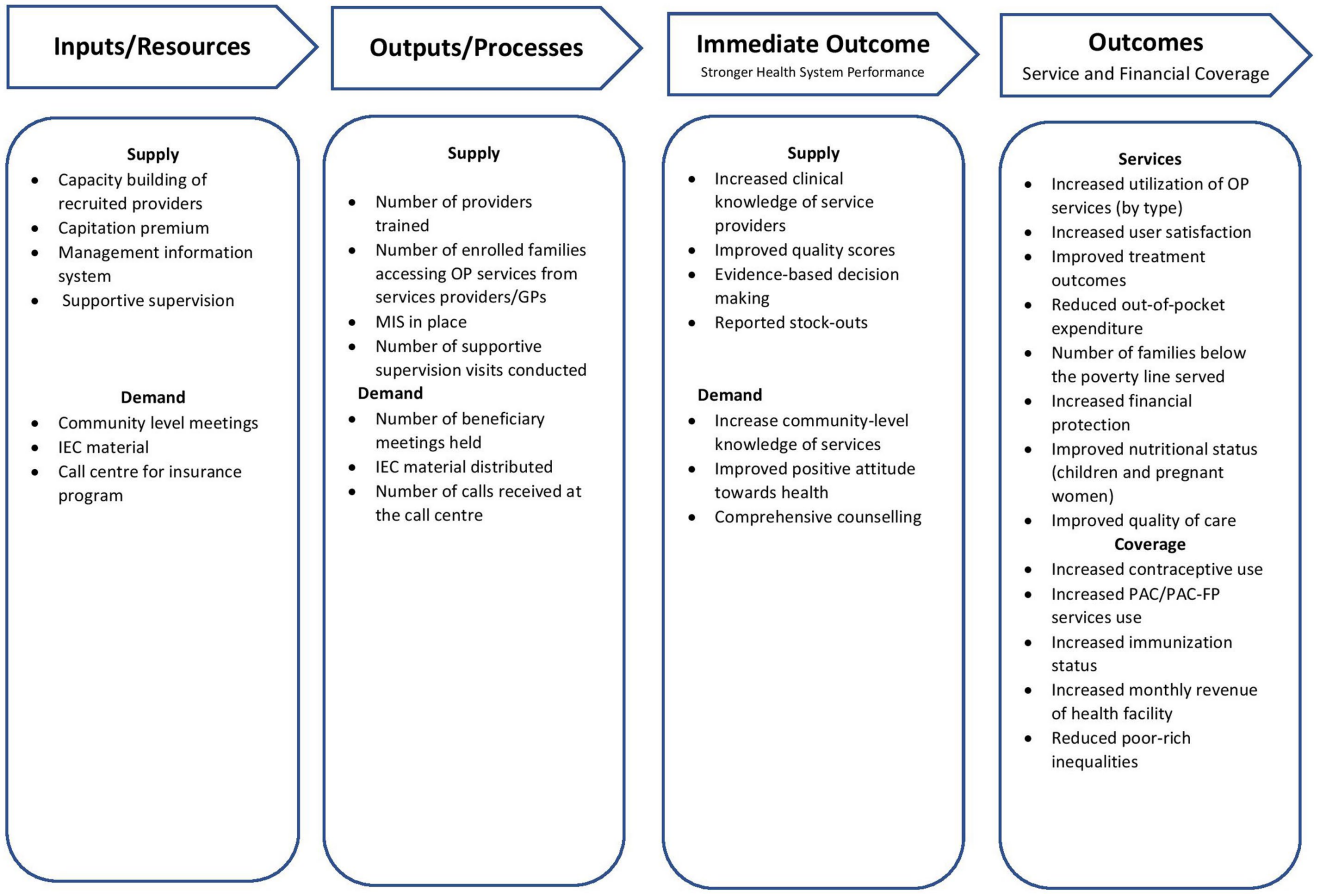
The logical framework.

The proposed study’s conceptual model will have three components (i) the Theory of Change (Figure 1), (ii) the Logical Framework (Figure 2), and (iii) the Capitation-based Provider Payment Mechanism (PPM). The theory of change gives the big picture and summarizes work strategically, while a logic model will illustrate the program’s (implementation) level of understanding of the change process. In other words, the logic framework will use a microscopic lens that zooms in on a specific pathway within the theory of change. The capitation is a prospective payment unit per family (per month or year). A payer makes a fixed payment for a defined set of services, regardless of the number of services provided.

#### The theory of change

The assumption is that access to primary health care services will increase by deploying private general practitioners (GPs) in select areas where public sector-owned primary-level facilities are unavailable. Three components of the theory of change are (a) the Demand Side, (b) the Supply Side, and (c) Evidence generation.

The supply-side and demand-side components will be combined while health financing using capitation for PHC may create demand, thereby increasing the utilization of PHC services for the poorest of the poor outpatients. Evidence will be generated by documenting the extent to which the needs of the beneficiaries are met with quality, equitable, sustainable PHC service and if this has redefined health-seeking behavior at the community level. Evidence will also provide an understanding of the suitability of the intervention with possible recommendations for health financing and its scalability. Each component has a defined set of activities/interventions to achieve the desired output, immediate, intermediate, and ultimate outcomes (Figure 1). From an individual and household perspective, the ultimate outcome is designed to reduce out-of-pocket expenditure while increasing immunization status, contraceptive use, financial protection, and treatment effects among the enrolled beneficiaries (refer to Figure 1). While at the health facility level, this project emphasizes the delivery of quality health service by enhancing the clinical and counseling skills of health service providers, resulting in increased client flow and revenue for the self-suitability of the service delivery channels.

#### The logical framework

The proposed framework will describe the process of evaluating the quantitative outcomes of health care costs on families covered by health insurance schemes who receive OPD services (intervention arm) as compared to those covered under SSP but do not receive OPD services (control arm). Figure 2 summarizes inputs, processes, or outputs leading to intended outcomes.

The logical flow of the theory of change emphasizes the intermediate and immediate outcomes of improving the attitude toward health-seeking behavior of the beneficiaries based on the Information, Education and Communication (IEC) material and awareness about health services. While the capacity building of the service providers will increase client-centric communication, clinical monitoring, client exit interview surveys, and routine data gathering, its usage will ensure quality health service delivery and improvement. This intervention has been designed to address the financial barrier that restricts access to primary health care services. Hence, this project is expected to improve access and uptake, reduce out-of-pocket expenditure for quality outpatient health services at the PHC level, and produce evidence for recommendations and their scalability.

#### Capitation-based provider payment mechanism (PPM)

For this research study, a proposed capitation payment mechanism will be implemented as a test case within the country’s existing health systems due to the desire to contain the cost of care provision. Literature indicates that capitation is expected to promote efficient use of resources by controlling prices and volumes of services provided. Capitations have been used both as stand-alone or blended capitations in large parts of Europe, Thailand, South Korea etc. (44) which not only help in managing cost and serve as a critical source of income for the providers (45) but are also associated with increased adherence to policies and general guidelines on the part of providers (46). In this research study, recruited providers will receive a fixed payment to provide a pre-defined benefit package of EPHS to each enrolled family allocated to their facility over a fixed period of 12 months. The implementing partner (Marie Stopes, Pakistan) will disburse the provider capitation fee on a four (4) quarterly payment cycle.

To determine the best capitation mechanism, this study proposes to test and evaluate a quarterly capitation fee totaling PKR 2,900 (USD 17.55) (47) paid to service providers per family per year. The amount for capitation was decided based on essential services selected for the implementation of this research study. The essential services were derived from Pakistan’s UHC benefit package at the PHC level. Payment of capitation fees will be independent of service provision and will not depend upon all or a fraction of enrolled families utilizing the services.

### Study design

This research study will use a mixed method, two arms, prospective, quasi-experimental controlled before and after (CBA) design. The intervention group will include beneficiary families with existing in-patient coverage under SSP who will receive OPD services as part of the research study. In contrast, the control group includes families with existing inpatient coverage under SSP but will not receive OPD services.

#### Study area

The study will be conducted in four (4) union councils (UCs) of ICT of Pakistan. Intervention and control arms will be assigned based on UCs selected for the study. The following are criteria for UC identification: (1) UC with sufficient residents of existing SSP beneficiaries, (2) UC in rural classified areas of ICT, (3) UC in which there is no public sector PHC level facility such as a Basic Health Unit (BHU), and presence of a network of private sector managed registered General Practitioners (GPs) providing primary healthcare services within identified UCs.

#### Study population and sample size

The target population will be divided into (a) demand-side and (b) supply-side.

*Demand Side:* The total sample size for this study is 1726 beneficiary families (Baseline and end-line: control and intervention). This has been calculated by assuming the overall prevalence of health outcomes to be 50% (conventional) at baseline and an anticipated 10-percentage point change because of the intervention; with 80% power and 0.05 significance level, the total estimated sample was 388 for each study arm. The estimated sample was adjusted for a design effect 2 to account for facility-level clustering and 10% missing data / non-response. Hence an overall sample size of 863 families in the intervention- and 863 families in the control- group will be required to test the study objectives. Families in each study arm will be selected randomly from the available list of SSP beneficiary families from four peri-urban Union Councils of Islamabad Capital Territory (ICT). The intervention group (*N* = 863) will receive modified EPHS interventions as OPD services, while the Control group (*N* = 863) will not receive OPD services. Enrolled families in the intervention group will be informed about the project, and regular follow-up of SSP outpatient services accessed will be undertaken.

*Supply Side:* GPs who are members of Marie Stopes Pakistan’s primary healthcare franchise network will be selected based on their service provision status within the UCs identified for research study implementation. Individual and facility assessments will be done to ascertain the capacity to provide quality OP services to clients. A training curriculum will be jointly developed by the MoNHSRC, SSP, MSS, and WHO and will be used to train the selected GPs on the modified EPHS services. Periodic quality assurance visits and a centralized data-collection management information system will be implemented.

#### Inclusion and exclusion criteria

Families with existing in-patient coverage of SSP, having SSP cards, and currently residing within the intervention or control areas of selected UCs of ICT will be considered eligible for inclusion in the research study and the baseline survey. Families not yet registered as beneficiaries with SSP and married children of SSP card holders were excluded. The endline survey will also follow similar inclusion–exclusion criteria as the baseline, i.e., to be conducted on those beneficiaries who were enrolled for the research study in the intervention and control UCs and those who were refusals or migrated cases will be excluded from the endline survey. The eligibility of each beneficiary family will be re-verified before inclusion through the National Database and Registration Authority (NADRA) website and by sending a Computerized National Identity Card (CNIC) number to 8,500 via short message service (SMS).

#### Study duration

The total duration of this research study is 24 months, with the following phases:

6-month inception period.

12 months research intervention period.

A 3-4-month period is dedicated to endline evaluation.

##### The benefits package - modified essential package of health services (EPHS)

The intervention group will receive the outpatient EPHS developed for the primary health care level, including sexual and reproductive health (48, 49) at the selected facilities, which include 10 broad categories of essential services with mandatory services and some referral services.

A list of the broad categories of EPHS are as follows:

**Table 1:** 

Reproductive Health, including modern contraceptives and post-abortion care	Childcare
Delivery Care (only low-risk BEmONC; otherwise, all referrals to higher-level facilities)	Adolescent health
Antenatal care	Infectious diseases
New-born care	Non-communicable diseases
Post-natal care	Basic tests and medicine (only approved for this study)

### Quantitative household surveys

Before initiating the intervention, a household-based baseline survey is proposed that will cover not only the study population but also a subset of the general population from the same catchment who do not have the provision of any public or private sector health insurance. Findings will explore and explain health-related expenses and payment mechanisms if practiced or that exist other than the fee for service paid through OOP by clients in the study catchment area. The baseline survey will assess the prevalence of study indicators, document socio-economic and demographic information, out-of-pocket health expenditure, and document KAP regarding existing inpatient coverage and access and uptake of health services. A household-based endline survey at the end of 12 months of the intervention will help assess study outcomes – the impact of the outpatient services model on knowledge, attitude, and practice regarding health insurance, access, and uptake of outpatient services at the PHC level, user satisfaction, treatment outcomes, OOP health expenditure and financial protection.

Study participants for baseline and end-line assessment across the intervention and comparison arm will be selected randomly from the list frame of the SSP. Using quantitative and qualitative techniques, data collection methods will include a population-based household survey, providers’ assessment, and specific qualitative inquiry during the intervention, routine services, client satisfaction, and monitoring (clinical quality assessment) data will be done similarly to a previous study using the same design (50).

### Qualitative inquiry

Using exploratory qualitative design, we will conduct interviews/focus groups before and after the intervention. The interviews will be conducted with the target audiences, which include beneficiary families, service providers, health managers/administrators, and policymakers. The overall purpose will be to explore perceptions and attitudes toward insurance schemes. During the baseline quantitative survey (or pre-intervention period), a needs assessment exercise will be conducted using a qualitative approach. The interviews will be aimed at understanding the community’s perceptions of health insurance in general and then specifically on the presently used (inpatient) package and newly designed outpatient package at the PHC level. For example, the following themes will be explored using 5–6 focus group discussions with the intervention beneficiary families:

- Utilization – explore the need for these services.
- Access – explore how you access these services; difficulty in accessing services skilled PHC level healthcare.
- Affordability – explore the ease of accessing these available services in your area. If you are unable to afford these services, how do you pay or how much can you pay for these services.
- Existing indoor coverage – explore the benefits and consequences of current benefits and satisfaction.
- Inputs will be specifically garnered on the design of the package, services covered, mechanism for reimbursement, and frequency of payments.

In addition, in-depth interviews with all service providers will be conducted in the catchment areas to identify the services with the highest perceived utility for end-users and programmatic approaches for price-setting, reimbursement, and payment methods best suited for service providers. Findings will be used to refine and finalize the outpatient services model and undertake detailed financial modeling if required before rollout. Moreover, 6–10 key informant interviews will be conducted with policymakers/insurance experts/program managers (health economists, public health professionals, etc.) with experience implementing health insurance in Pakistan. Input from experts will be used further to inform the final design of the insurance model to explore the utility of adaptive learning. The use of evidence in social health protection initiatives to incorporate outpatient services programming using a benefits package integrated Health Insurance (using capitation payments) and to ascertain progress at the public sector policy-making level concerning project recommendations. At the time of the endline survey (around the post-intervention period), a qualitative stocktaking exercise will be implemented to explain the following themes from beneficiaries’ and service providers’ viewpoints and gauge their experiences:

- Experience with the Programme.
- Impact on the Community.
- Recommendations for Future Programs.

In addition, we will specifically explore facilitators and barriers encountered by service providers during the intervention period. We will also seek their suggestions to improve the integrated health financing model. The team of researchers, including the principal and co-investigators, will develop the interview guides for beneficiary focus group discussions, service provider in-depth interviews and key-informant interviews during the project inception period by the researcher.

#### Data collection and management

Baseline and endline surveys, including qualitative inquiry, will be conducted by a 3rd party consultant/organization. Enumerators with prior experience will be hired and trained on theoretical and practical aspects of the health insurance programs, study instruments, research ethics, and interviewing techniques. The field supervisor will conduct shadow interviews (observation of data collection) and independent re-interview/assessment of study participants (10% of participants). All data will be collected electronically on tablets. The software applications will have built-in validation checks to minimize data errors. All software applications will be tested before their use in the study. The implementing partners will undertake routine service delivery statistics, clinical monitoring of the quality of care, and client satisfaction.

#### Data analysis

The quantitative data analysis will focus on the combined effect of the complete package of interventions and the outcome(s) rather than exploring each component. Quantitative data will be analyzed through SPSS Software using descriptive and inferential statistics. The main outcome variables will be increased access, uptake, satisfaction, contraceptive prevalence, and reduction in OOP expenditure. Difference-in-differences (DiD) estimation will compare key outcome variables between the intervention and control group pre- and post-intervention (51). It will also measure the intervention effect on any subgroups in the intervention/control groups by comparing key outcome variables between beneficiaries in the intervention and control groups.

**Figure 3:**
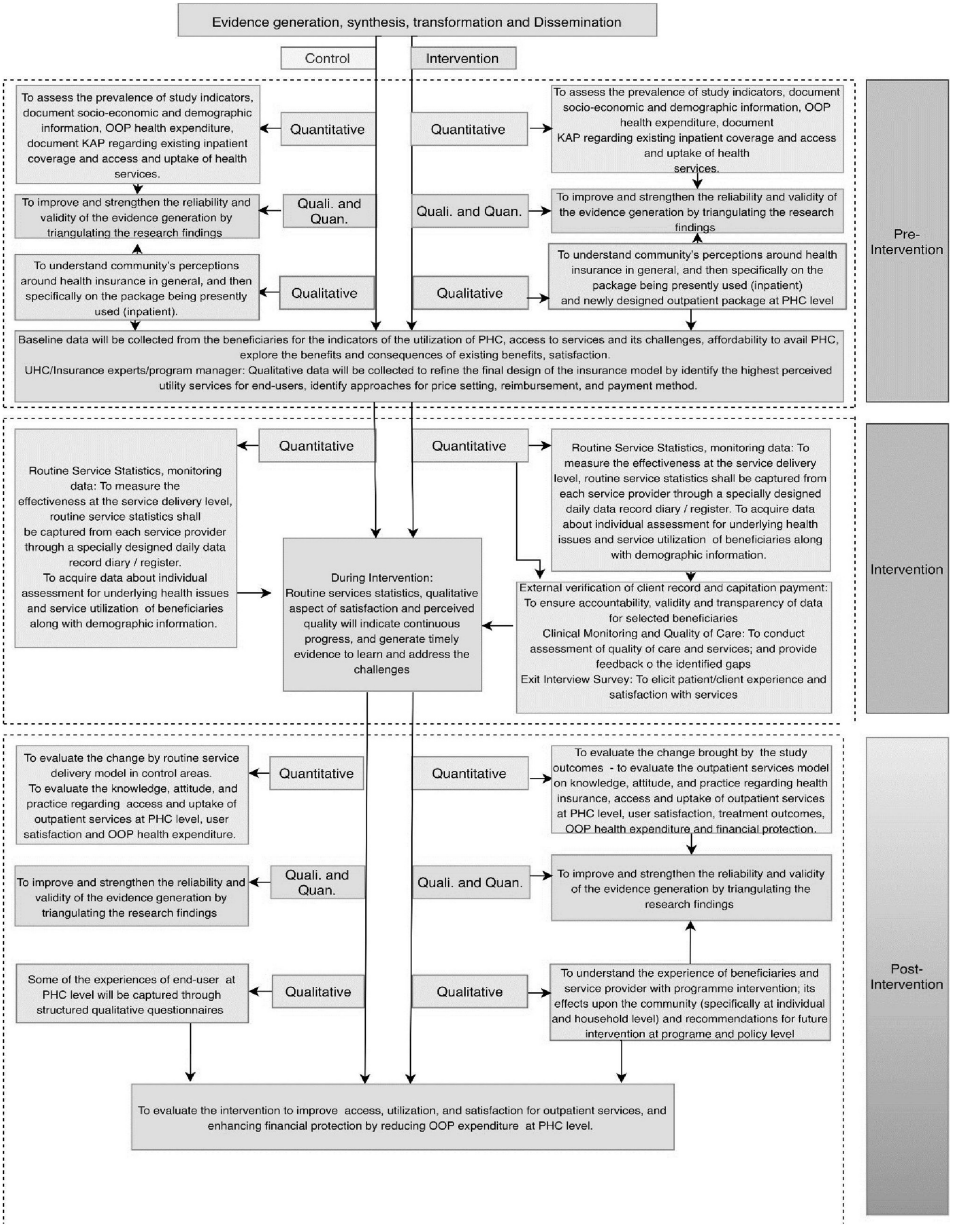
The evidence synthesis plan.

For the qualitative data, content and thematic analysis will be done in Nvivo Software version 11. Codes will be developed after reading the transcripts without losing the context. For qualitative analyses, transcripts will be read several times to understand the respondents’ in-depth experiences and views. ‘Meaning units’ that mirror statements will be identified as per the topic guide by highlighting phrases in the transcripts, which will be ‘condensed’, and after that ‘, codes’ will be identified from the ‘condensed meaning units’ without losing the context. Finally, the research team will review the codes independently and group similar codes into sub-categories and categories. From the categories, themes and sub-themes will be identified after systematically analyzing the commonalities, variations, and disagreements. We will further analyse data with a focus on the description and interpretation of message meaning and concepts to validate the study’s findings. Quantitative and qualitative data will be triangulated to provide comprehensive information about the impact of the research intervention. Figure 3 describes the components of the data synthesis plan envisaged for the project.

### Ethics and dissemination

The study received formal ethical approval from the Research and Development Solutions, Pakistan-Institutional Review Board (IRB00010843). Preserving the highest standards of ethics in research and practice is critical. Hence, actions will be taken to ensure the highest standards of ethics for the quality of collected data. Informed written consent will be taken from all research participants in their native language. No personal identifying information will link recorded data to a specific individual. Steps will also be taken to ensure privacy (both auditory and visual) during interviews. Unique IDs will be issued to each participant based on the home address and allocated PHC facility so that the names of any participant might not be disclosed. The same unique identifier will identify all data forms, reports, and other records to maintain participant confidentiality. All the data documents will be kept in the custody of authorized personnel in locked cabinets and shared only with authorized personnel. Participants will also be notified of their rights to withdraw from the study at any time if they so choose, as well as their right to skip any questions during baseline/endline assessment if they do not feel comfortable answering without facing any penalties. A core group will be represented by the Ministry of Health Government of Pakistan, MSS-P, SSP, and WHO which will be responsible for programmatic decision-making. At the initial design phase, this core group will finalize and approve the modified EPHS to be used in the research intervention, jointly develop a training curriculum for the GPs and finalize the amount for capitation based on the modified EPHS for this research study. Findings emerging from this research study will be disseminated through peer-reviewed journal publications, presentations at relevant conferences, and shared with key stakeholders engaged in this project, including government officials and the World Health Organization.

## Discussion

People living in LMIC, including in Pakistan, often do not benefit from primary health care programs for early detection and treatment of people with risk factors for multiple diseases; the poorest are the most affected. Around 50% of the population in Pakistan does not have access to primary healthcare services, and a similar proportion of the population does not have health insurance, which seems to contribute to poverty due to catastrophic health spending and high out-of-pocket expenditure. Evidence suggests that emphasis should be placed on primary health care to achieve universal health coverage. The private sector can be a major provider of essential services where the public sector has capacity (human resources/coverage/finances) constraints. Therefore, the proposed research study is expected to address the gap in the SSP program by engaging private sector service providers for outpatient services at the primary healthcare level to reduce the out-of-pocket expenditure of the already enrolled beneficiaries, thus improving universal health coverage. Finally, it will help to develop a blueprint referral system to reduce unnecessary hospital admissions and improve timely access to healthcare. Expanding the reach of basic primary healthcare services is crucial to improving health and providing ready access to basic healthcare services, including sexual reproductive health services under primary care, can reduce hospitalizations, morbidity, and mortality, increase life expectancy, and deliver better overall health.

### Possible limitations and strengths

Generally, quasi-experimental pre- and post-intervention designs are research methods that lack the random assignment of participants to groups, which is a characteristic of true experimental designs. But in our study, the beneficiary families in each study arm (intervention and control) will be selected randomly from the available list of Sehat Sahulat program beneficiary families from the four (4) peri-urban/rural Union Councils of Islamabad Capital Territory (ICT). However, the intervention and control of private-sector-owned primary healthcare facilities will be selected conveniently in the areas where similar primary healthcare public sector facilities are unavailable, and there is a scarcity of established private-sector PHC facilities. Nonetheless, the pre-post intervention design with control is internationally accredited when controlled experimental trials are not feasible due to logistic, financial, or other ethical reasons. While these designs have their strengths, they also come with limitations. Here are some considerations; for example, the quasi-experimental designs allow researchers to study interventions in natural settings. We will ensure no spillover within different intervention areas by choosing areas at a minimum distance from each other. The difference in cultural background of participants from the intervention and control areas can become a potential limitation. However, since the intervention and control areas are located within the same city, we believe the differences would be minimal and, consequently, have a limited impact on the study findings. Another potential limitation can be the presence of competing health providers providing similar primary health care services operating within the areas of project health providers. Selecting a similar healthcare facility for the project where no other service providers exist is difficult. To address this limitation, we will use the control group to assess the impact of routine practice in health facilities toward primary healthcare services, including family planning and sexual reproductive health services, anticipating that any increase in outcomes in our study will be due to the project intervention(s). We will conduct evaluation activities by hiring an external consultant and informing participants about the study’s purpose and confidentiality before collecting data. During regular monitoring from the research team, some random visits will be made to check the reliability of data.

The validity is often compromised in quasi-experimental designs; however, our study will use statistical techniques and control measures to strengthen its validity, such as developing a study protocol as a standardized way to conduct the study to prevent researcher, instrumentation, or testing biases. When everyone follows the specific procedures, you can be more confident that the results will be valid. We will carefully select the control facility and justify the choice of the control group, ensuring that the control group is comparable to the intervention group in terms of relevant characteristics. A matched pre-post design would have been a stronger study design to compare behaviors of the same sample, as it will allow controlling for unobserved confounders such as to help address selection bias issues, control baseline differences and improve group comparability. Through Difference-in-Differences (DiD) analysis, we will compare outcome changes between the intervention and control groups, accounting for pre-existing trends. This will help control external factors that may affect both groups similarly. We will conduct validity checks for balance in covariates between groups before and after matching or weighting procedures. We will also provide clear and transparent reporting of methods, measures, and results to enhance the credibility and replicability of the study. In addition, respondents may have faced difficulties in answering questions or recall bias that captured information about their past experiences, such as information provided by the service provider about side effects at the time of method uptake, decision making and source of referral to the health facility or where the contraceptive method was adopted, exact time and discontinuation of method etc. Hence, whenever possible, we will try to supplement self-reported data with objective measures to validate the information provided by participants. Or to use standardized instruments and validated questionnaires to enhance the reliability of data collection.

## Conclusion and knowledge contribution

Primary healthcare (PHC) plays a pivotal role in a nation’s healthcare infrastructure, acting as the initial interface for patients and delivering clinical treatment at the community level. Strengthening primary healthcare requires transitioning from a narrow focus on treating specific diseases to providing personalized, holistic, and consistent care that addresses the needs of both patients and communities, thereby decreasing dependence on professional healthcare services. The effectiveness of primary care is significantly enhanced when access to higher levels of care and services is coordinated through gatekeeping, such as referrals from primary care, or when there are financial incentives for seeking care at the primary level, like minimal or no out-of-pocket payments. Such arrangements can also improve continuity of care and ensure that specialized services are utilized optimally within the healthcare system rather than being excessively utilized for conditions that can be appropriately managed in primary care settings, thus improving overall efficiency. Therefore, primary and community health services investments are essential to ensure access to the most cost-effective interventions. However, in many countries, patients either do not seek help from or bypass primary healthcare facilities due to issues such as poor accessibility (e.g., distance to facilities and treatment costs), low quality of care (e.g., lack of clinician competence and pharmaceuticals), and weak gatekeeping mechanisms. Notably, insufficient spending on primary and community health services is often seen as a major factor contributing to inequities and inefficiencies in healthcare. Consequently, urgent attention from policymakers is crucial for integrating routine primary healthcare and establishing sustainable indigenous healthcare financing mechanisms. The proposed research study aims to explore and study a service delivery model which harnesses the private sector to deliver an essential package of health services, including sexual and reproductive health services as outpatient services under SSP, ultimately facilitating UHC. Findings will provide a blueprint referral system to reduce unnecessary hospital admissions and improve timely access to healthcare. A robust PHC system can improve population health, lower healthcare expenditure, strengthen the healthcare system, and ultimately make UHC a reality.

## Ethics statement

The study received formal ethical approval from the Research and Development Solutions, Pakistan Institutional Review Board (IRB00010843).

## Author contributions

SA: Conceptualization, Data curation, Formal analysis, Funding acquisition, Investigation, Methodology, Project administration, Resources, Software, Supervision, Validation, Visualization, Writing – original draft, Writing – review & editing. ET: Conceptualization, Data curation, Formal analysis, Funding acquisition, Investigation, Methodology, Project administration, Resources, Software, Supervision, Validation, Visualization, Writing – original draft, Writing – review & editing. MAr: Funding acquisition, Investigation, Project administration, Resources, Supervision, Validation, Visualization, Writing – review & editing. HH: Conceptualization, Data curation, Formal analysis, Investigation, Methodology, Software, Supervision, Validation, Visualization, Writing – original draft, Writing – review & editing. AA: Conceptualization, Methodology, Project administration, Supervision, Writing – review & editing. AB: Conceptualization, Project administration, Supervision, Writing – review & editing. MAw: Conceptualization, Investigation, Methodology, Project administration, Software, Supervision, Visualization, Writing – original draft, Writing – review & editing. FR: Conceptualization, Funding acquisition, Investigation, Methodology, Project administration, Resources, Software, Supervision, Validation, Visualization, Writing – original draft. NH: Data curation, Investigation, Validation, Visualization, Writing – review & editing. UQ: Conceptualization, Data curation, Funding acquisition, Investigation, Methodology, Project administration, Resources, Supervision, Validation, Visualization, Writing – review & editing.
